# Can ChatGPT and Gemini justify brain CT referrals? A comparative study with human experts and a custom prediction model

**DOI:** 10.1186/s41747-025-00569-y

**Published:** 2025-02-18

**Authors:** Jaka Potočnik, Edel Thomas, Dearbhla Kearney, Ronan P. Killeen, Eric J. Heffernan, Shane J. Foley

**Affiliations:** 1 University College Dublin School of Medicine, Dublin, Ireland; 2 Mater Misericordiae University Hospital, Dublin, Ireland; 3 St. Vincent’s University Hospital, Dublin, Ireland; 4 Royal Victoria Eye and Ear Hospital, Dublin, Ireland

**Keywords:** Artificial intelligence, Brain, Radiology, Referral and consultation, Tomography (x-ray computed)

## Abstract

**Background:**

The poor uptake of imaging referral guidelines in Europe results in a substantial amount of inappropriate computed tomography (CT) scans. Publicly available chatbots, ChatGPT and Gemini, offer an alternative for justifying real-world referrals. Recent research reports high ChatGPT accuracy when analysing American College of Radiology Appropriateness Criteria variants. We compared the chatbots’ performance in interpreting, justifying, and suggesting alternative imaging for unstructured adult brain CT referrals in accordance with the European Society of Radiology iGuide. Our prediction model for automated iGuide categorisation of referrals was also compared against the chatbots.

**Methods:**

The iGuide justification of 143 real-world CT brain referrals, used to evaluate a prediction model, was analysed by two radiographers and radiologists. ChatGPT-4’s and Gemini’s imaging recommendations and pathology suspicions were compared with those of humans, with respect to referral completeness. Inter-rater reliability with κ statistics determined the agreement between entities.

**Results:**

Chatbots’ performance was limited (κ = 0.3) but improved for more complete referrals. The prediction model outperformed the chatbots in justification analysis (κ = 0.853). The chatbots’ interpretations of complete referrals were highly consistent (49/52, 94.2%). The agreement regarding alternative imaging was high for both complete and ambiguous referrals, with ChatGPT and Gemini correctly identifying imaging modality and anatomical region in 83/96 (86.5%) and 81/96 (84.4%) cases, respectively.

**Conclusion:**

The chatbots’ ability to analyse the justification of adult brain CT referrals is limited to complete referrals, unlike our prediction model. Further research is needed to confirm these findings for other types of CT scans and modalities.

**Relevance statement:**

ChatGPT and Gemini exhibit potential in justifying free text brain CT referrals; however, further improvements are required to handle real-world referrals of varying quality.

**Key Points:**

- Custom prediction model’s justification analysis strongly aligns with iGuide and surpasses chatbots.
- Chatbots incorrectly justified almost one-half of all CT brain referrals.
- Chatbots have limited performance in justifying ambiguous CT brain referrals.
- Chatbot performance improved when referrals were detailed and included suspected pathology.

**Graphical Abstract:**

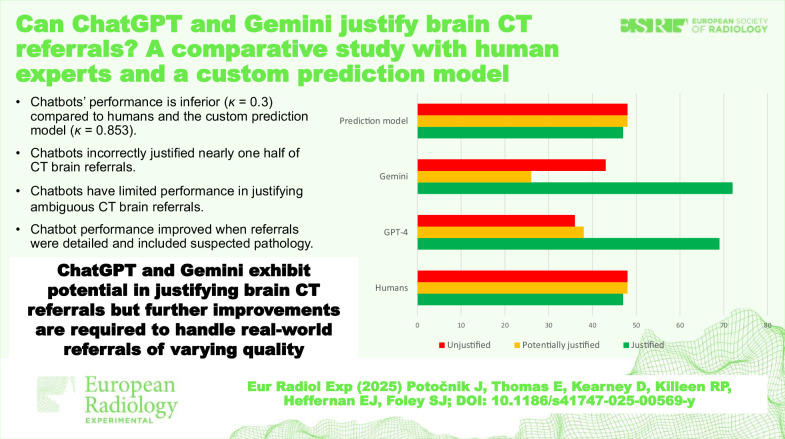

## Background

The proliferation of generative artificial intelligence (AI) has led to the development and availability of numerous large language models (LLMs) to the public, also known as “chatbots”. For instance, Chat Generative Pre-Trained Transformer (ChatGPT) by OpenAI (San Francisco, CA, USA) [1], and Gemini by Google (Google Inc, Mountainview, CA, USA) [2] are just two among many. The two conversational AI models are designed to understand, interpret, analyse, and generate human-like text based on the input received.

Although diagnostic imaging referral guidelines are required by the European 2013/59/EURATOM Basic Safety Standards Directive [3], existing evidence suggests poor adoption within European institutions and suboptimal application of computed tomography (CT) justification audits [4–7]. Using imaging referral guidelines can improve the appropriateness of imaging by approximately 20% [8]. Therefore, it is reasonable to consider alternative approaches, such as AI, for the efficient implementation of clinical imaging guidelines. This is even more important for CT, which contributes the highest collective effective dose among all imaging modalities [9].

There is an increasing amount of research investigating the potential clinical decision support (CDS) role of ChatGPT in radiology, including in justification. Currently, the chatbot can recommend diagnostic imaging for several clinical presentations in line with the American College of Radiology Appropriateness Criteria [10], including breast cancer screening [11], certain thoracic and abdominal pathologies [12, 13], and specific neurologic presentations [14]. However, most of these studies [11, 13, 14] involved prompts derived from the Appropriateness Criteria variants, which are more structured, and of higher quality compared to the clinical text typically found in radiology referrals and can be found online. Another study demonstrated high ChatGPT consistency with the European Society of Radiology (ESR) iGuide [15] when recommending diagnostic imaging for patients with abdominal symptoms based on free text referrals [16]. A slightly different yet promising approach to auditing CT referrals involves machine learning and deep learning for automated iGuide categorisation of unstructured CT referrals [17]. The best-performing classifier (a bag-of-words-based gradient-boosting classifier) outperformed clinical staff when auditing free text adult brain CT referrals.

While the aforementioned studies demonstrate the tremendous potential and impact that LLMs could have on radiology-referring practice, it is important to note that neither study considered whether ChatGPT’s decisions and suggestions regarding alternative imaging are supported by its suspicions about the underlying condition. Furthermore, a strong preference for using only ChatGPT in this type of research has been noted. As a result, we aimed to address these limitations by including Gemini and by comparing the two chatbots with human experts and the best-performing prediction model for justifying the most common CT referral nationally, adult brain CT [18]. Additionally, we sought to determine how the completeness of referrals impacted the justification analysis with LLMs.

## Methods

All manually annotated anonymised adult brain CT referrals from the test set (*n* = 143) used in our previous study [17] to evaluate machine learning- and deep learning-based prediction models for classifying the justification of brain CT referrals in accordance with iGuide were included. Since the anonymised retrospective dataset was used, the study was granted an ethics exemption by our institution’s research ethics committee (REERN: LS-E-21-216-Potocnik-Foley).

The test set was part of a larger dataset used in our previous study, where a retrospective justification audit of approximately 3,000 adult brain CT referrals sourced from three Irish CT centres was performed. The outcomes of the audit served as target features for training and testing various supervised models for classifying referrals. Random downsampling to the minority class was performed to address the issue of class imbalance. This yielded 714 referrals, 238 per class. These were randomly split into stratified training and test sets (80/20), of which 571 were used to train prediction models. The remaining 143 referrals were used to evaluate various prediction models and now the two chatbots. The best-performing model was the bag-of-words-based gradient boosting classifier, achieving nearly 95% accuracy and a macro F1 of 0.94. The model’s hyperparameters were tuned with 10-fold cross-validation on the training set through GridSearch. The associated text preprocessing pipeline tokenised free-text clinical indications into individual words. Our stop-word filter removed both English and non-clinical stop words, except for negations. Common abbreviations were then spelt out, and any misspelt words were corrected with the Enchant algorithm. Any errors in the Enchant’s outputs were manually mapped to the correct terminology. All data science tasks were performed in Python (version 3.8.16).

### Establishing a reference standard

Two radiographers (6 years and 8 years of experience) manually analysed each referral by matching its unstructured clinical indication with the ESR iGuide structured indication of choice. Referrals with disagreements were subsequently analysed by two radiologists (11 years and 15 years of experience). A final decision regarding the justification analysis of referrals reviewed by the radiologists was based on either a majority vote or consensus among the four readers in the case of a tie. For unjustified and potentially justified referrals, an alternative imaging method was proposed. Since data providers request generic brain CT for related referrals, *i.e*., non-contrast CT, contrast-enhanced CT, CT angiography, etc., the justification and alternative imaging were considered in the context of modality and anatomical region.

The first author (J.P.) analysed each referral individually to determine its completeness based on how detailed clinical indications were (*i.e*., complete or ambiguous). A referral was deemed ambiguous when it was subject to multiple interpretations and lacked information on suspected pathology and/or a primary concern. Otherwise, a referral was deemed complete.

### Prompting LLMs

ChatGPT-4, available through a paid subscription, and the freely available Gemini were used. Both LLMs were blinded to the scan type and the results of the human analysis. Unstructured prompts, derived from the original dataset, were utilised to compare various interpretations between human experts and LLMs. The phrase “Identify the two most appropriate diagnostic imaging examinations based on the following unstructured indication specified in a diagnostic imaging referral: *Free text*” was added to the LLM input to collect data on justification and alternative imaging. To confirm each LLM’s understanding of unstructured indications and their decisions regarding the most appropriate imaging, the second prompt, “What is the most likely pathology that this patient is suffering from?” verified their suspicions. Since the custom prediction model can process thousands of referrals within seconds without manual input, the time required to manually prompt and receive responses from each LLM was measured across five referrals and then averaged. Prompts and referral text were copied and pasted for each instance.

Outputs from ChatGPT-4 and Gemini were collected between March 20 and 21, 2024. A single chat session was used for each referral to avoid the influence of past LLMs’ answers on their decision-making. Since the human experts identified iGuide structured indications rather than disease processes, the matching of these is rare and limited to cases where the iGuide indications are diseases. For such referrals, where a chatbot identified a pathology that may be consistent with the human experts’ selection of iGuide structured indications, the chatbot’s interpretation was deemed consistent with that of human experts. This approach was adopted rather than requesting the two chatbots to provide structured iGuide indications for each referral. Upon initial prompting, we found that the LLMs hallucinate and provide false indications when asked to do so. For instance, when ChatGPT was asked to identify the corresponding iGuide structured indication for ‘*81 y/o presenting with sudden onset left lower limb weakness with no other neurological symptoms. Also complains of intermittent episodes of fuzziness in the head. Past medical history of vertigo*.’, its response was ‘Weakness—acute, unilateral’. This structured indication cannot be found in the iGuide database. Likewise, Gemini falsely concluded that ‘Acute Neurological Syndrome’ is the corresponding iGuide indication for the same referral. Alternative prompting requesting to ‘associate the free text with iGuide referral guideline variants’ or ‘match the free text with iGuide referral guidelines’ resulted in false outputs as well.

The output of each LLM, which is in free text format, was individually analysed by the first author (J.P.) to identify data on justification, interpretation, and alternative imaging. Upon initial prompting, the first author and the radiologists agreed to assign importance to the order of diagnostic imaging methods provided, along with the accompanying explanations clarifying whether the imaging method was considered the first choice or supplementary/alternative. Then, the radiologists validated the first author’s analysis by randomly selecting and reviewing one-third of referrals (*n* = 45) and indicating whether they agreed with the justification and pathology interpretations. Sample prompts, LLMs’ responses, and the author’s interpretations of those are included in Table 1. If the LLM did not consider brain CT at all, the referral was deemed unjustified.

**Table 1:** LLMs’ responses regarding the most appropriate diagnostic imaging for sample referrals and the author’s interpretation of these

Prompt	ChatGPT-4 response	Gemini response	Authors’ interpretation
Identify the two most appropriate diagnostic imaging examinations based on the following unstructured indication specified in a diagnostic imaging referral: “HEAD INJURY, GENERALISED TONIC CLONIC SEIZURE, GCS 12/15. LEFT TEMPORAL LACERATION ? INTRACRANIAL INJURY. NO PREVIOUS HISTORY OF SEIZURE”.	1. Non-contrast head CT Scan: this is the first-line imaging modality for acute head injuries due to its wide availability, rapid acquisition time, and excellent sensitivity for detecting acute haemorrhage, skull fractures, and other emergent conditions that could explain the patient’s symptoms and GCS score. A non-contrast CT is preferred initially to avoid obscuring any bleeding with the contrast agent. 2. MRI of the brain: magnetic Resonance Imaging (MRI) provides superior detail of brain structures and is more sensitive than CT for detecting non-acute intracranial injuries, such as diffuse axonal injury, small contusions, or any ischaemic injuries. MRI is particularly useful for evaluating the extent of brain damage following the initial assessment and management of acute injuries. Given the patient’s seizure without a previous history of seizures, an MRI might be considered to identify subtle injuries not seen on CT, or post-acute changes, once the patient is stabilized.”	1. Head CT: this is a quick and readily available imaging test that can detect bleeding, fractures, and other abnormalities in the brain. It is often the first-line imaging study for patients with head trauma. 2. MRI brain: this imaging test provides more detailed images of the brain than a CT scan and can be helpful in identifying soft tissue injuries, such as contusions and shearing injuries. It can also be useful for evaluating epileptogenic foci (areas of the brain that are responsible for seizures).	Both ChatGPT-4 and Gemini deem brain CT justified.
Identify the two most appropriate diagnostic imaging examinations based on the following unstructured indication specified in a diagnostic imaging referral: “PATIENT HAD EPISODE OF SEVERE LIGHT HEADEDNESS THIS MORNING. RESOLVED IN 5 min”.	1. Brain MRI with or without contrast: an MRI of the brain is a highly sensitive tool for detecting a wide range of neurological conditions, including ischaemic stroke, which might not yet have resulted in permanent damage but could manifest as transient symptoms like lightheadedness. It can also identify other potential causes such as lesions or abnormalities in brain structure that could contribute to the symptoms. 2. Carotid ultrasound: this test is used to evaluate the carotid arteries in the neck, which supply blood to the brain. It can identify narrowing or blockages (stenosis) that could lead to transient ischaemic attacks (TIAs) or strokes, presenting with symptoms such as lightheadedness. It is a non-invasive, safe, and effective way to assess vascular health and the risk of cerebrovascular events.	1. MRI brain: this imaging study provides a detailed view of the brain and can help identify abnormalities that might be causing lightheadedness, such as stroke, tumour, or bleeding. 2. MRA head and neck: this imaging study examines the blood vessels in the head and neck and can help identify problems with blood flow that could be causing lightheadedness.	Both ChatGPT-4 and Gemini deem brain CT unjustified.
Identify the two most appropriate diagnostic imaging examinations based on the following unstructured indication specified in a diagnostic imaging referral: “PERSISTENT RIGHT TEMPORAL HEADACHE WITH NAUSEA, NO OTHER NEUROLOGY, EXAM NORMAL”.	1. MRI of the brain (without and with contrast): MRI is highly sensitive for detecting a wide range of brain pathologies, including tumours, vascular abnormalities, demyelinating diseases, and infectious processes. The use of contrast can help in further delineating lesions and assessing for any enhancing features suggestive of active pathology. MRI is preferred for its superior soft-tissue contrast resolution compared to CT, making it more effective in detecting subtle abnormalities. 2. CT scan of the head (without contrast): a non-contrast CT scan would be a quicker and more readily available test that can be very effective in ruling out acute haemorrhage, which is a critical concern in the setting of headache. While it is less sensitive than MRI for many types of intracranial pathology, it is a good initial imaging test, especially if MRI is contraindicated (*e.g.,* in patients with certain types of metal implants) or not immediately available.	1. MRI of the brain: this is the most appropriate initial imaging study because it provides detailed anatomical images of the brain and surrounding tissues, which can help identify potential causes of the headache such as tumours, vascular malformations, or inflammation. MRI is also highly sensitive for detecting abnormalities in the temporal lobe, which is the area of the brain associated with the patient’s right-sided headache. 2. MRV of the brain: this can be considered alongside the MRI scan. MRV specifically visualizes the brain’s venous system and can help identify abnormalities like blood clots or narrowing of veins that could be contributing to the headaches.	ChatGPT-4 deems brain CT potentially justified. Gemini deems brain CT unjustified.

*CT* Computed tomography, *GCS* Glasgow coma scale, *MRI* Magnetic resonance imaging, *MRV* Magnetic resonance venography

### Statistical analyses

Descriptive statistics were utilised to visualise the frequency distributions of the justification analyses, and quantify interpretation- and alternative imaging-related comparisons. Simple Cohen’s κ was calculated to determine the level of agreement between all entities regarding the justification of referrals in accordance with iGuide, including the validation of the first author’s interpretations.

## Results

**Fig. 1:**
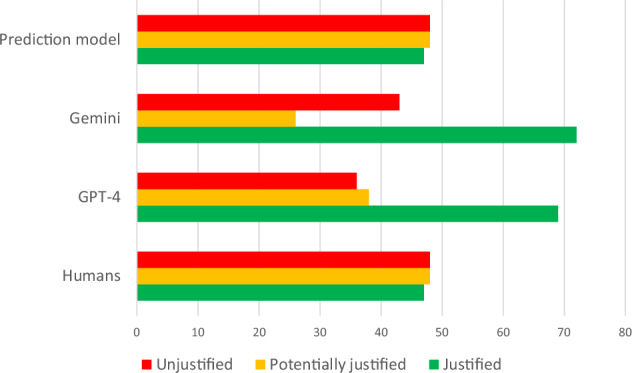
Frequency distributions of the brain CT justification analysis outcomes

The test set comprised 47/143 (32.9%) justified, 48/143 (33.6%) potentially justified, and 48/143 (33.6%) unjustified referrals previously analysed by human experts. In total, 91/143 (63.6%) referrals were ambiguous, of which only 11 (12.1%) were justified. 45/91 (49.5%) ambiguous referrals were unjustified, representing nearly all (45/48, 95.8%) unjustified referrals in the dataset. The radiologists confirmed the first author’s interpretations and identified no discrepancies between themselves and the first author, resulting in κ = 1.000. The average processing time per referral with LLMs corresponds to approximately 20 s. Frequency distributions visualising outputs of the human, chatbot, and the prediction model justification analyses can be seen in Fig. 1. In total, ChatGPT incorrectly justified 67/143 (46.9%) referrals, which is similar to Gemini’s 64/143 (44.8%) misclassified referrals. Gemini refused to analyse two referrals due to being “only a large language model” and “not having the necessary information or abilities”. The most noticeable difference was in the rate of justified referrals, where ChatGPT-4 and Gemini deemed 69/143 (48.3%) and 72/143 (50.3%) referrals justified, respectively, in contrast to humans and the prediction model (47/143, 32.9%). Gemini categorised only 26/143 (18.2%) referrals as potentially justified, which was less compared to humans, the prediction model, and ChatGPT-4 (38/143, 26.6%). The latter deemed only 36/143 (25.2%) referrals unjustified.

Table 2 illustrates pairwise justification agreements between different entities. The custom prediction model made only eight false predictions, achieving nearly perfect agreement (κ = 0.853) with the reference standard. While the agreement between the chatbots was moderate (κ = 0.547), their discrepancies from the reference standard are significant, with κ values of 0.330 and 0.353.

**Table 2:** Agreement levels between different entities regarding the justification analysis of brain CT referrals expressed in Cohen κ

Humans and ChatGPT-4	Humans and Gemini	Humans and prediction model
κ = 0.330	κ = 0.353	κ = 0.853

ChatGPT-4 and Gemini	ChatGPT-4 and prediction model	Gemini and prediction model
κ = 0.547	κ = 0.348	κ = 0.414

Out of 91 ambiguous referrals, ChatGPT-4’s justification decisions were consistent with those of human experts in 36 (39.6%) referrals, whereas Gemini agreed on four referrals more (40/91, 44.0%). On the other hand, for the remaining 52 referrals where previously missing information in ambiguous referrals was present, ChatGPT-4 and Gemini agreed with the human justification analysis in 40/52 (76.7%) and 39/52 (75.0%) cases, respectively.

Similarly, the amount of information provided in referrals influenced the interpretation of unstructured indications by LLMs. Both ChatGPT-4 and Gemini interpreted 36/91 (39.6%) ambiguous referrals identically to humans. A substantial increase in agreement was observed when interpreting referrals containing sufficient information, with each LLM agreeing with humans in 49/52 (94.2%) cases. Table 3 presents sample complete and ambiguous referrals along with interpretations and justification from each entity, excluding the prediction model.

**Table 3:** Sample unstructured clinical indications with associated interpretations and justifications from each entity

		Interpretations of unstructured indications			Justification
Sample referral text	Completeness	Humans	ChatGPT-4	Gemini	Humans/ChatGPT-4/Gemini/prediction model
“78-years-old male with hx of dizziness and hearing loss with unsteady walking. Hx head trauma 20 years ago. No visual symptoms or focal neurological deficit.”	Ambiguous	Dizziness/vertigo/inner ear pathology, hearing changes	Bilateral vestibulopathy	Stroke, tumours, intracranial haemorrhage	No/Yes/Yes/No
“Please assess for subdural, banged head 5/52, headache since then, left side, parietal, slight confusion, no blood thinners.”	Complete	Subdural haematoma, headache after trauma	Subdural haematoma	Subdural haematoma	Yes/Yes/Yes/Yes
“Left-sided headache, behind left eye, blurred vision yesterday.”	Ambiguous	Migraine	Optic neuritis	Migraine	Potentially/No/Potentially/Potentially
“22-year-old lady within last 2/52 had 3 ep of collapses last one this am with loc few seconds sustained lt frontal hi? Intracranial haemorrhage.”	Complete	Intracranial haemorrhage, traumatic brain injury	Intracranial haemorrhage	Intracranial haemorrhage	Yes/Yes/Yes/Yes
“Admitted with poor balance and progressive worsening vertigo abdominal distention. Having large volume ascites on ct abdomen and deranged renal functions.”	Ambiguous	Dizziness/vertigo/inner ear pathology	Liver cirrhosis	Liver disease	No/No/No/No
“C/O intermittent numbness to the tongue, lips and rt arm + hand. C/O intermittent weakness to rt hand, normal clinical examination? SOL? Demyelination”	Complete	Demyelinating disease with(out) spinal cord symptoms, primary or secondary neoplasm of the central nervous system	Demyelination—multiple sclerosis	Demyelination affecting tongue, lips, and right arm and hand	Potentially/No/No/Potentially

*C/O* Complains of, *CT* Computed tomography, *Hx* History of, *RT* Right, *SOL* Space occupying lesion

Regarding the suggestion of alternative imaging, Table 4 summarises the agreement rates in the context of imaging modality and anatomical region. Ambiguous unjustified referrals show the least agreement (75.6%) for both chatbots. Otherwise, the high agreement is noted across the board, with ChatGPT-4 making two more correct suggestions than Gemini.

**Table 4:** Chatbots’ agreement rates with the reference standard regarding alternative imaging modality and anatomical region for complete and ambiguous referrals

	Complete referrals		Ambiguous referrals	
Chatbot	Potentially justified	Unjustified	Potentially justified	Unjustified
ChatGPT-4	11/12 (91.7%)	3/3 (100.0%)	35/36 (97.2%)	34/45 (75.6%)
Gemini	10/12 (83.3%)	3/3 (100.0%)	34/36 (94.4%)	34/45 (75.6%)

## Discussion

In this study, we evaluated the performance of ChatGPT-4, Gemini, and a custom prediction model in justifying adult free-text brain CT referrals with reference to the ESR iGuide. A previously conducted manual audit of referrals involving four human experts served as the reference standard. Additionally, we assessed ChatGPT’s and Gemini’s ability to interpret unstructured indications and suggest alternative imaging for potentially justified and unjustified referrals.

Considering the justification analysis, a perfect κ score of 1.000 would indicate 100% agreement with human experts. κ scores of 0.330 and 0.353 were achieved for ChatGPT-4 and Gemini, respectively, indicating that the performance of the LLMs is far from that of humans. Specifically, both chatbots struggled to justify ambiguous referrals, which lacked clinically relevant details, such as suspected pathology or specific concerns necessary for determining appropriate diagnostic imaging. Our study highlights this important limitation, and its findings correlate with a similar study concluding that a human clinician significantly outperformed ChatGPT in selecting appropriate neuroimaging [14]. In contrast, the justification analysis tended to be consistent and showed high agreement when the suspected pathology or concern was specified in the referral. In our previous study, humans were not perfectly consistent in analysing the justification of referrals either, demonstrating that CDS in the form of online guidelines is insufficient and does not guarantee structured, consistent interpretations and overall analysis [17]. In contrast, our custom prediction model trained on 571 adult brain CT referrals sourced from three clinical sites was able to capture the underlying patterns within the dataset and achieved strong agreement with humans.

The LLMs’ ability to interpret free-text referrals also depends on the completeness or integrity of the information provided. Strong agreement with humans for more detailed or specific referrals suggests that the chatbots based their justification decisions on correct or at least similar assumptions. Conversely, poor agreement indicates that a substantial portion of referrals was misinterpreted, suggesting that the initial decision-making process regarding appropriateness was based on these misinterpretations. For example, all entities agreed that the second-to-last referral in Table 3 is unjustified, but for different reasons. In contrast, considering the very last referral in the table, all entities interpreted the referral in a nearly identical way. However, the chatbots’ decision regarding the appropriateness was inconsistent with iGuide. This highlights another important limitation of LLMs when determining the appropriateness of neuroimaging. On a positive note, our findings suggest that, where appropriate, the LLMs are likely to identify the correct alternative imaging modality and anatomical region to be scanned based on the free text in referrals. It is important to note that chatbot prompting was done in English, and non-English prompts may result in different outputs and overall findings.

In general, the issue of suboptimal, incomplete referrals has not been effectively addressed, raising the ethical concerns surrounding patient care in radiology departments [19]. When considering the clinical potential of chatbots, the ability to accurately process referrals of varying quality is vital, particularly when no structured system is in place. While ChatGPT is not intended for CDS, OpenAI offers the option to create and use custom GPTs, which could be of interest to a wider research community in the context of justification. Care needs to be taken when uploading custom datasets to ensure ethical and legal compliance. An alternative is to train custom prediction models using local or, in the best case, national datasets. It would be reasonable to consider developing an LLM specific to radiology referring practices and using it in combination with machine and deep learning for classifying referrals with respect to their quality, compliance with the ESR iGuide, and more.

With most parts of the EU AI Act [20] becoming applicable in 2026, there will be very little space available for black-box models in healthcare, such as the two chatbots, as their decision-making process is not transparent, and their outputs are unexplainable [21]. Ideally, the chatbots should provide evidence-based decisions with clear reference to guidelines. As these guidelines are likely to evolve, both custom prediction models and LLMs should be able to improve iteratively.

At the moment, the LLMs are unable to link unstructured clinical indications with structured ESR iGuide indications, even when multiple prompts are provided. Instead, they hallucinate and provide false indications, which is problematic for clinical use. When such limitations are encountered, prompt engineering may not yield the desired output. However, it may shed light on previously unknown LLM limitations. Since ChatGPT and Gemini developers are not emphasising their utility and explainability in radiology, the scientific community has less control in steering the development of these models—unless the process of training a custom GPT becomes radiology-oriented, among other areas.

On the contrary, when developing a custom model for analysing referrals sourced locally or nationally, model development is controlled and can be configured to learn and adapt over time based on inputs and enabled human interactions. A combination of a chatbot and a custom prediction model could be considered when developing CDS for justifying diagnostic imaging. For example, the chatbot could facilitate communication between the user and the custom prediction model.

AI has the potential to address the entire lifecycle of a referral through prospective and retrospective CDS by facilitating real-time support in requesting and reviewing referrals, as well as enabling efficient retrospective audits. With its objective, guideline-driven assistance, AI could lessen the impact of well-known barriers currently limiting the adoption of guidelines, including defensive medicine or the fear of ‘missing something,’ inconvenience of access during busy periods, and lack of awareness, among others. By automatically auditing referrals as they are received and retrospectively, AI can reduce the cognitive load on radiologists and radiographers. This can be done efficiently only when the AI-enabled solution is integrated with electronic health records, eliminating any additional manual workload. Care needs to be taken to prevent over-reliance and ensure that AI outputs are critically evaluated against evidence-based knowledge.

There are limitations to our study. First, it included only adult brain CT referrals, the majority of which were ambiguous. Therefore, the findings of our study cannot be generalised to other types of CT scans and imaging modalities. Second, considering the usage of contrast media or specific imaging protocols was unfeasible due to the generic ordering practices at participating sites. The specific characteristics of each referral (*e.g*., urgency, patient type, other contextual metadata) were not provided, so certain aspects of the LLM and prediction model generalizability were not investigated. In light of this, further prompting to conduct a detailed error analysis to identify specific weaknesses in the LLMs, reasons why specific decisions were made, and factors contributing to misinterpretations might be of interest. Third, LLMs are continuously being improved, making ChatGPT-4 and Gemini dated as of May 2024 (*e.g*., ChatGPT-4o, Gemini Advanced/2.0 Pro). Fourth, the custom prediction model was trained on limited local data, making comparisons with state-of-the-art LLMs on a completely external and larger dataset unfeasible. Lastly, different LLM prompts yield different outcomes; by instructing each LLM to provide the two most appropriate imaging examinations, it was more feasible to determine the categorisation of each referral. Should the LLMs be allowed to list three or more imaging examinations, categorisation would have become more challenging, complex, and subject to more than one interpretation.

In conclusion, we observed a limited chatbot performance in justifying ambiguous referrals, which is not the case for complete referrals. Subject to further improvements in processing clinical text, LLMs have the potential to assist in interpreting and justifying radiology referrals or determining appropriate diagnostic imaging. Future research needs to consider using real-world referrals of varying quality from other types of CT scans and imaging modalities to challenge chatbots appropriately.

## Data Availability

The datasets used and/or analysed during the current study are available from the corresponding author upon reasonable request.

## Abbreviations

AI: Artificial intelligence
CDS: Clinical decision support
ChatGPT: Chat generative pre-trained transformer
CT: Computed tomography
ESR: European Society of Radiology
LLMs: Large language models
